# Supplementary material for: Carbon nanotube dry adhesives with temperature-enhanced adhesion over a large temperature range
Source: Nat Commun. 2016 Nov 16;7:13450. doi: 10.1038/ncomms13450 (PMC5116088; doi:10.1038/ncomms13450)
Supplement: Supplementary Information — Supplementary Figures 1-18, Supplementary Tables 1-3, Supplementary Discussion and Supplementary References. [file ncomms13450-s1.pdf]

Supplementary Figures

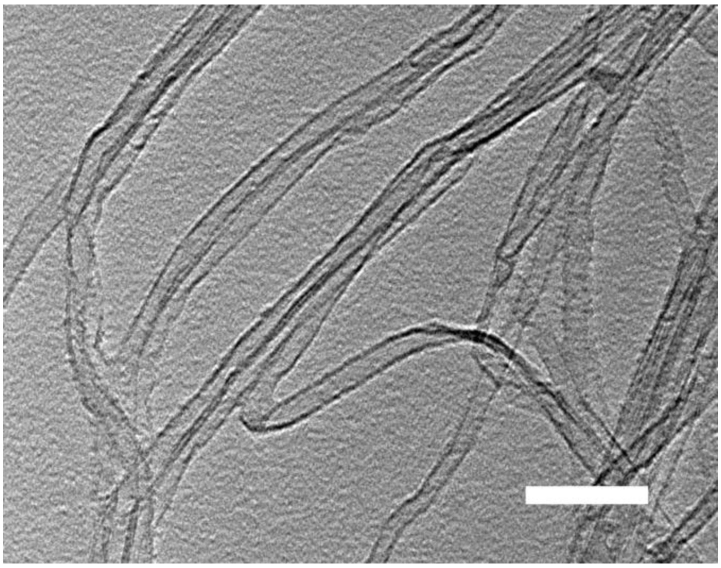

Supplementary Figure 1 | Tube structure of carbon nanotube (CNT) adhesive. A typical high-resolution transmission electron microscopic (TEM) image showing that double-walled CNTs dominate the CNT dry adhesive with the average outer diameter of 7-10 nm (scale bar: 50nm).

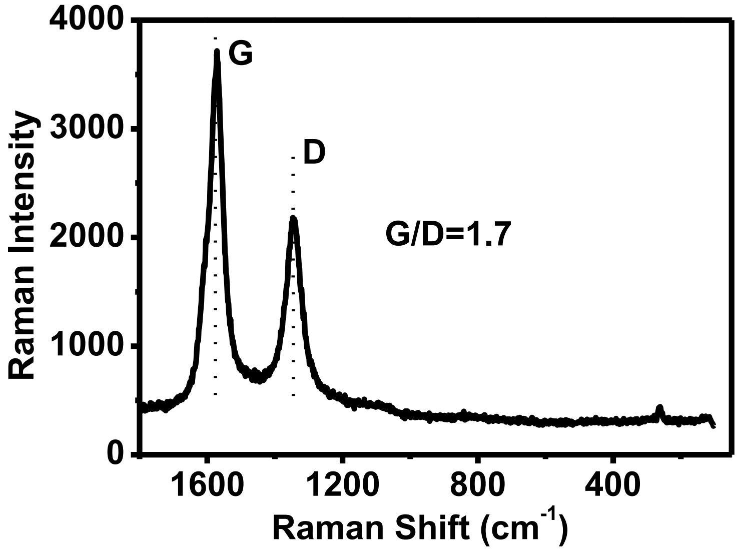

Supplementary Figure 2 | Raman characterization of CNT adhesive. Raman spectrum excited by 532-nm wavelength showing the G/D ratio of 1.7.

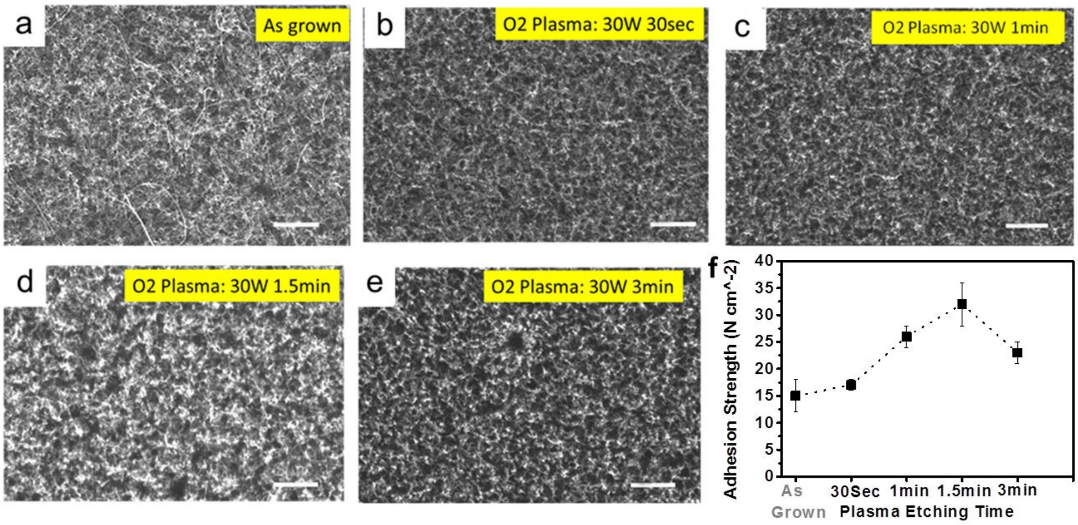

Supplementary Figure 3 | Optimization of plasma treatment condition. a-e, Top-view SEM images of the CNT dry adhesives demonstrating the adjustment of micro-level spacing of the nodes by tuning the plasma treatment conditions (scale bar: 2 µm). **f,** Dependence of the adhesion strength on the oxygen plasma etching time under plasma power of 30 W, frequency of 63 Hz, and monomer pressure of ~1 Torr at ambient temperature. Each of the adhesion strength data points was averaged from five tested samples subjected to plasma etching under the same conductions. The variation of the error bars seen in **(f)** is most probably related to the roughness mismatch between the substrate and the plasma-etched CNT adhesives with the longer error bar for the bigger roughness mismatch.

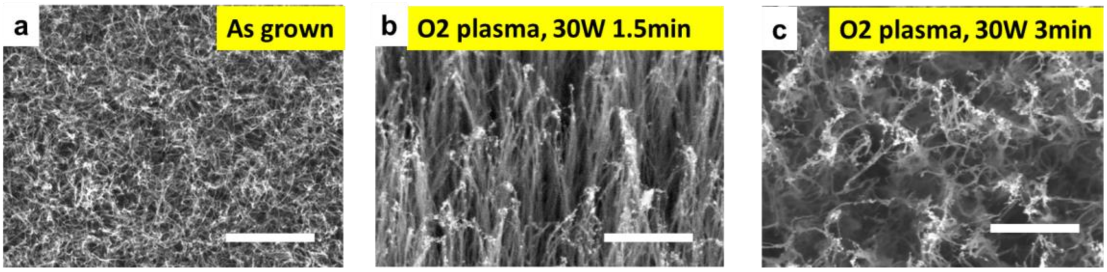

Supplementary Figure 4 | Enlarged views showing the plasma etching effect. FE-SEM images (10K magnification) showing the effect of plasma etching on the CNT array structure (scale bar: 1μm). The plasma etching initially removed the nonaligned nanotube segments with the concomitant top node formation to cause the nanotube bundling. However, over etching could cause the collapse of the nanotube bundle with decreased adhesion strength (*cf.* Supplementary Figure 3f).

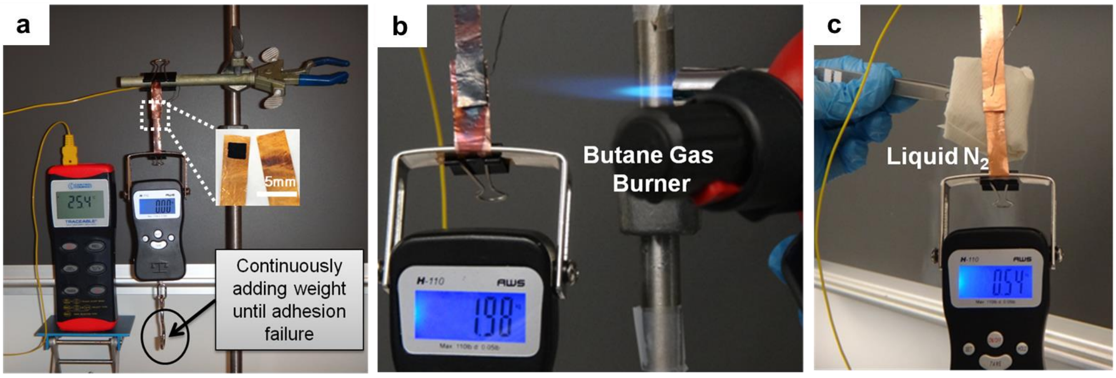

Supplementary Figure 5 | Set-up for dry adhesion measurements. a, 25.4°C. **b,** 1033°C. **c,** -190.7°C (*cf.* Methods in the main text).

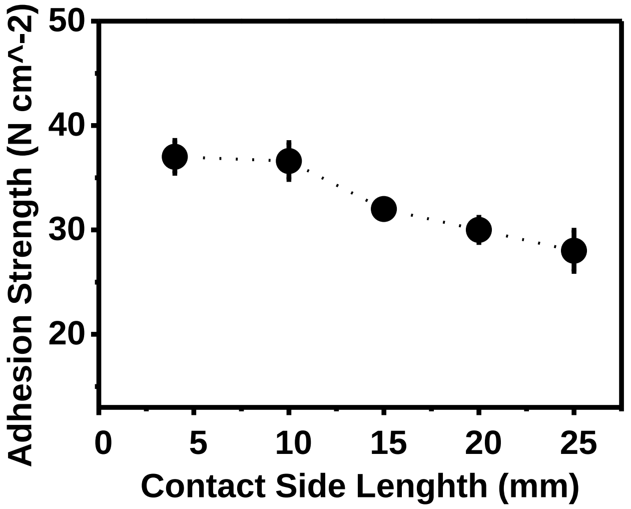

Supplementary Figure 6 | Effect of contact area on adhesion strength. Dependence of the adhesion strength on the contact side length of square CNT adhesive samples, showing the relationship between the adhesion strength and the adhesive contact area. The slight decrease in the adhesion strength with the increasing adhesive contact area up to 2.5×2.5 cm^2 are supposed to be resultant from the non-uniform CNT height grown at larger area, giving the poorer contact (*cf.* Methods in the main text). Each of the adhesion strength data points was averaged from five samples of the same sample size. The error bars represent the whole range of the measured values for each of the five sample groups.

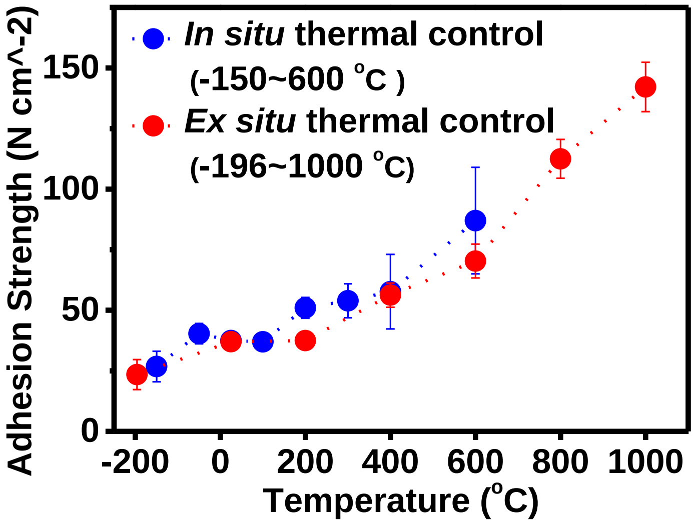

Supplementary Figure 7 | Adhesion measurements under *in situ* and *ex situ* thermal control. Adhesion strength measured from the CNT dry adhesive being thermally treated with an *in situ* (heated and adhesion measurement in TA Instruments, RSA-G2 with Environmental Test Chamber) and *ex situ* (heated inside of a muffle furnace and cold down to room temperature for the adhesion force measurements) temperature control. Each of the adhesion strength data points was averaged from five tested samples under the same thermal control conductions. The variation of the error bars is caused by the temperature fluctuation and the system error.

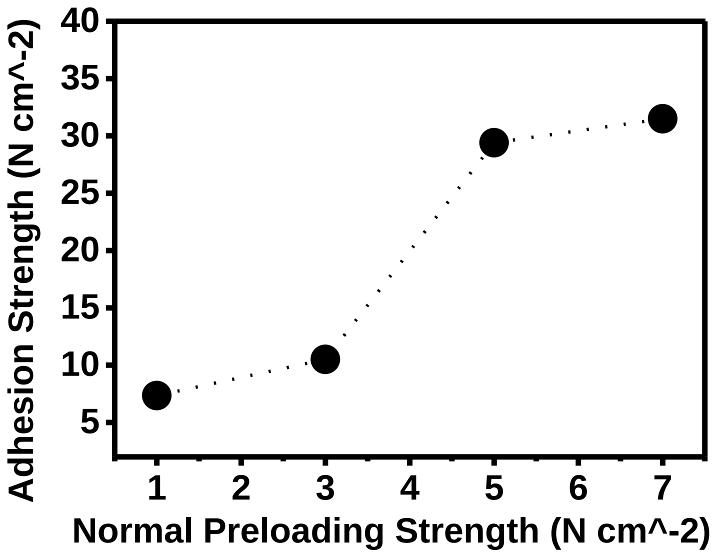

Supplementary Figure 8 | Effect of normal preloading on the adhesion. Adhesion strength measured as a function of normal preloading strength during the attachment procedure.

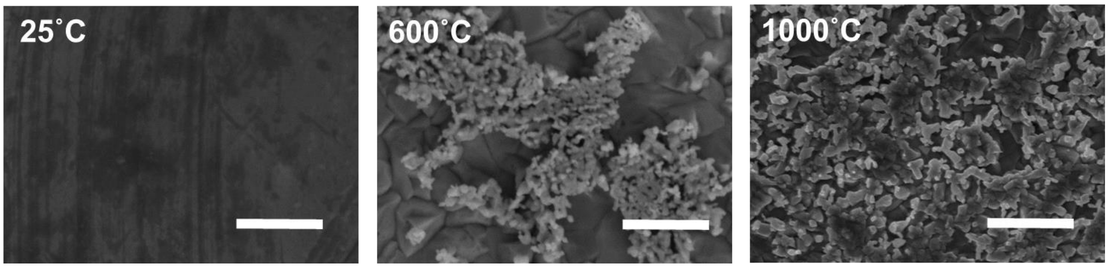

Supplementary Figure 9 | Surface morphologies of copper foils at various temperatures. SEM images showing the occurrence of copper surface segregation and roughening when annealed at high temperatures (scale bar: 1μm).

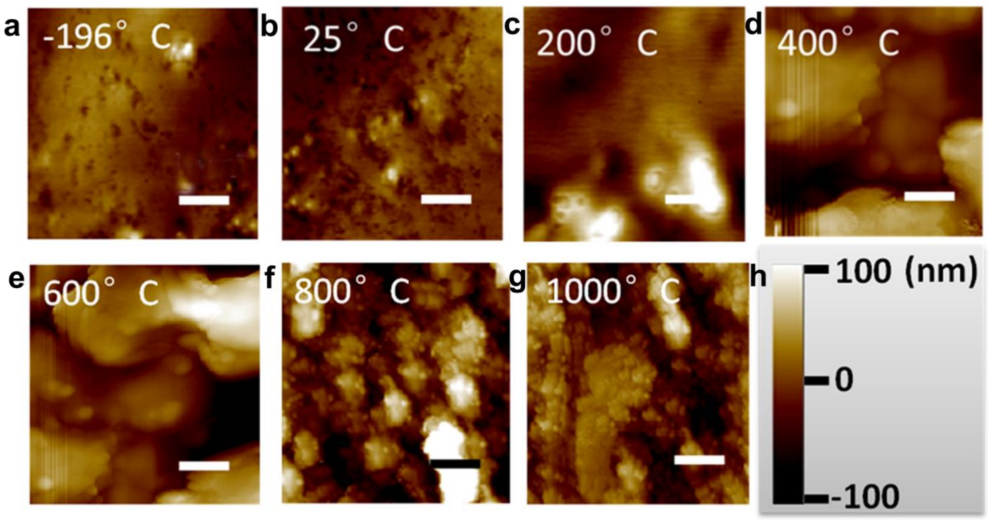

Supplementary Figure 10 | Surface roughness of copper foils at various temperatures. a-g, AFM images showing the increased roughness of copper surfaces with increasing temperature (scale bar: 1μm). **h,** Height scale of AFM images.

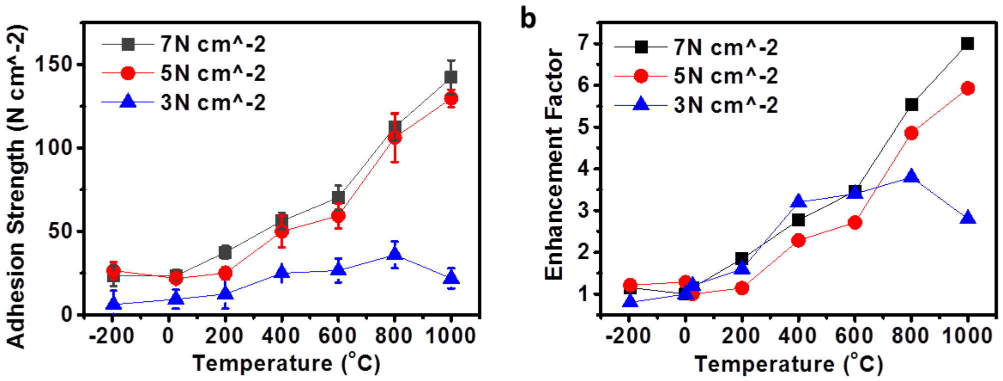

Supplementary Figure 11 | Effect of preloading strength on the thermal enhancement of adhesion. Temperature dependence of **(a)** adhesion strength and **(b)** enhancement factors (the ratio of the adhesion strength measured at a specific temperature to that at room temperature) of the CNT adhesive from -196 to 1000°C measured on the CNT adhesives with a preloading strength of either 3 N cm^{-2}, 5 N cm^{-2} and 7 N cm^{-2}. Each of the adhesion strength data points was averaged from five samples pre-pressed by the same normal loading force and tested under the same temperature condition. The error bars represent the whole range of the measured values for each of the five sample groups.

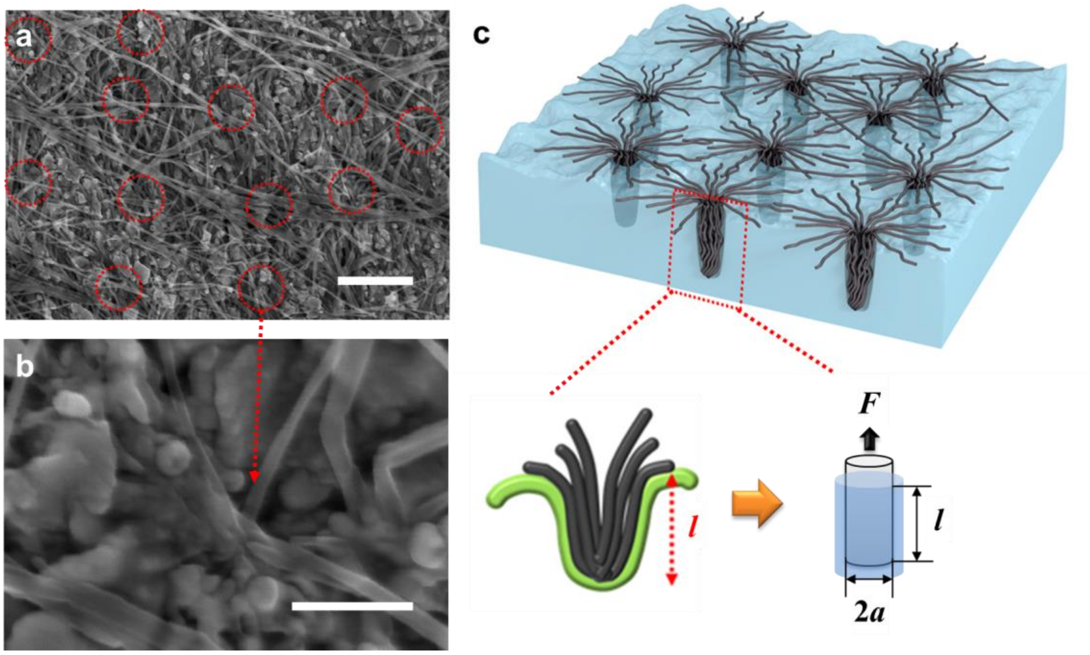

Supplementary Figure 12 | Nano-interlocking enhancement model. **a-b,** SEM observation on the embedded node-guided CNT bundles penetrated deeply in the cavities at high temperature (1000°C), illustrating the screw-like nano-interlocking adhesion strengthening (scale bar: (a) 1μm; (b) 0.2μm). **c,** The individual CNT bundle was modeled as an elastic strand to be embedded in a surface asperity with the depth of *l* and radius of *a* as investigated by SEM observation.

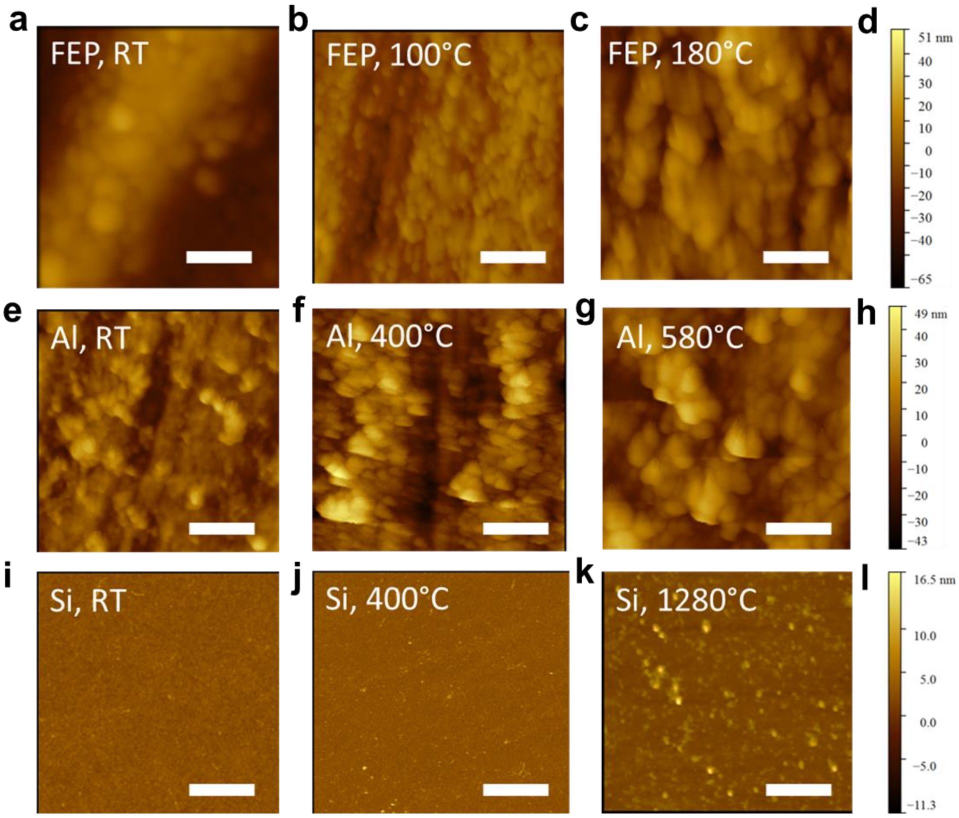

Supplementary Figure 13 | Surface roughness of fluorinated ethylene propylene (FEP) film, aluminum (Al) foil, and silicon (Si) wafer at various temperatures. AFM images showing the increased roughness of FEP film **(a-c)** and Al foil **(e-g)** with increasing temperature. **d**, The corresponding height scale for **(a-c)**. **h**, The corresponding height scale for **(e-g)**. **i-k,** AFM images showing the temperature-insensitive hard smooth surface of Si wafer. **l**, The corresponding height scale for **(i-k)**. (Scale bar: 0.5μm)

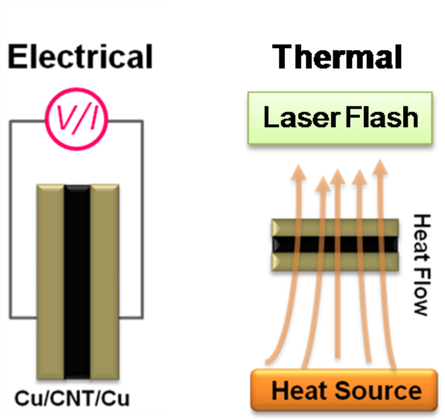

Supplementary Figure 14 | Electrical and thermal measurements. Schematic representations of the test set-ups for the electrical and thermal measurements.

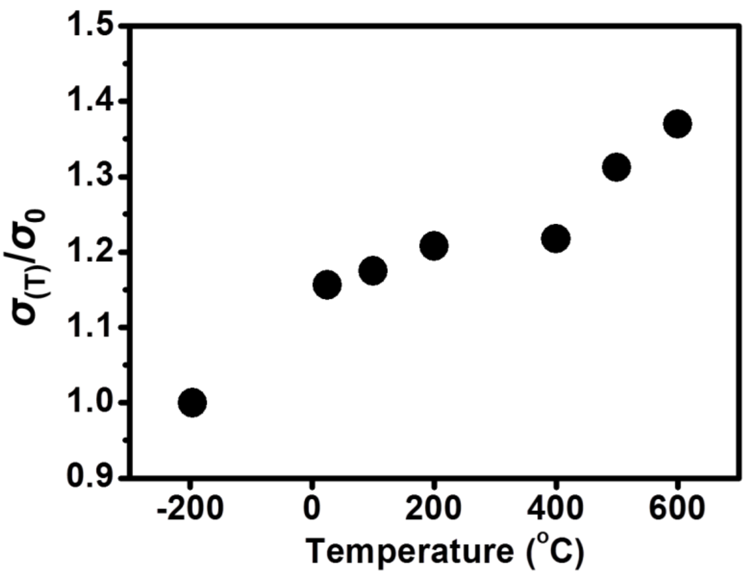

Supplementary Figure 15 | Temperature dependence of relative electrical conductivity. The conductivity at the specific temperature ($\sigma_{(T)}$)/conductivity at liquid N_2 temperature of the pristine CNT array (σ_0) showing an increase in conductivity with increasing temperature, which is revisable.

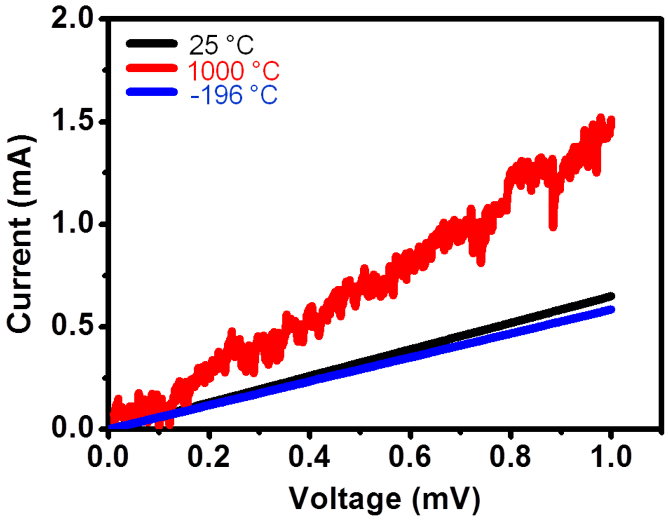

Supplementary Figure 16 | Temperature-invariant contact mode between the Cu and CNT adhesive. Linear *I-V* curves measured at 25ºC, -196ºC and 1000ºC, showing an Ohmic contact between the CNT dry adhesive and Cu surface.

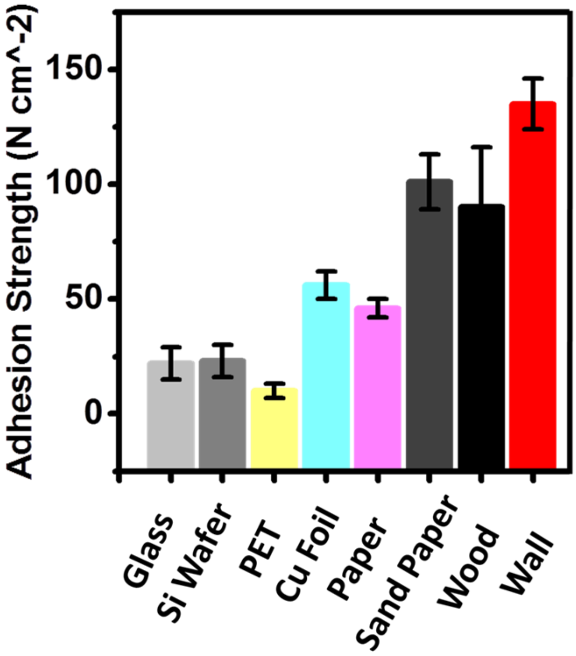

Supplementary Figure 17 | CNT adhesion on rough surfaces. Adhesion strengh of the CNT dry adhesives aganist various naturally rough surfaces, including metal foils, polymers, wood pieces, paper sheets, and even painted walls, with the adhesion force ranging from 37 to 137 N cm^{-2} (Supplementary Movie 1) at ambient temperature. Each of the adhesion strength data points was averaged from five samples tested under the same condition. The error bars represent the whole range of the measured values for each of the five sample groups. The variation of the error bars is most probably related to the mismatch between the microstructure of CNT surface morphology and roughness of substrates.

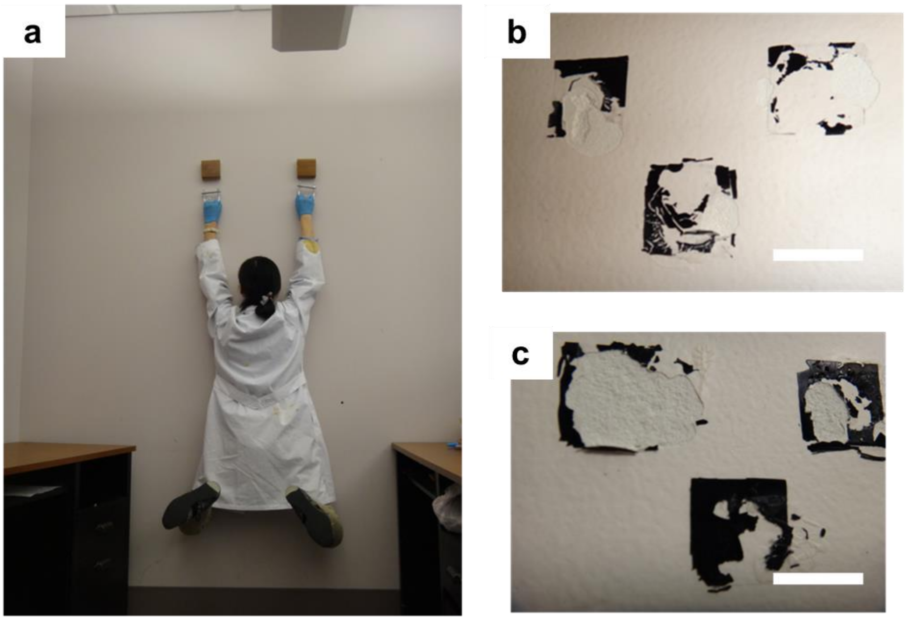

Supplementary Figure 18 | Demonstration of dry adhesion on painted wall. a, A human being of 54kg by weight was held on the painted wall by using 6 pieces of the 2 cm×2 cm CNT adhesives supported by two wood handles (Supplimentary Movie 2). **b-c**, The paint wall was partially peeled off by the three pieces of the CNT dry adhesives after deliberately taking each of the two wood handles away from the wall, indicating a very strong adhesion of the 2 cm×2 cm CNT adhesives to the wall (scale bar: 2cm).

Supplementary Tables

Supplementary Table 1. Comparison of the VA-CNT adhesive developed in this work with others previously reported.

Vertically aligned CNTs	Adhesion strength (N cm^-2)	Substrate	Temperature Range	Reference
Multiwall	100 (shear)	100N/cm^2 on Glass *<10N/cm^2 on rough surfaces	RT (room temperature)	1
	10 (normal)			
Single wall	29 (shear)	Glass (smooth)	25~216°C	2
	15 (normal)			
Multiwall & Doublewall mix (Pattern)	36 (shear)	Glass (smooth)	RT	3
Multiwall	11.7 (shear)	Si Substrate (smooth)	RT	4
	7.8 (normal)			
Multiwall (Pillar)	7.8 (shear)	Si Substrate (smooth)	RT	5
Multiwall	6 (normal)	Si Substrate (smooth)	RT	6
	26 (shear)			
Double wall	143(shear)	Various surfaces (e.g. wall (137N/cm^2), wood (101N/cm^2), paper (48N/cm^2)) *Roughness enhanced adhesion	Thermally enhanced adhesion of temperature cycle from -196 to 1000°C more than 400 cycles	This work

Supplementary Table 2. Table of the estimated values of Rq for asperity features from the AFM investigation (Supplementary Fig. 10) on the target copper surfaces at different temperatures.

Temperature [°C]	Roughness (Rq) [nm]
-196	10.5
25	9.8
200	25.4
400	59.3
600	92.7
800	147.3
1000	177.3

Supplementary Table 3. Comparison between CNT adhesive and Epoxy permanent adhesive**

Adhesive Type	Attachment Mode	Adhesion strength (N/cm²)	Repeatability	Attachment Process	Temperature Range (° C)	Target Surface	Electrical and thermal properties
CNT Adhesive	Dry Adhesion /vdW interaction	37~145	Repeatable; Reduced to 30% the 2nd round	Pressure sensitive, preloading required	-196~ 1033	Versatile: glass, metal, polymer	Electrically and thermally conducting
Conventional Permanent Glues	Wet chemical crosslinking interaction	Up to ~2500	Not repeatable	Cure, heat/pressure required	< 100	Surface selective	None

**http://multimedia.3m.com/mws/media/1054052O/acrylic-adhesives-recent-advancements-white-paper.pdf
http://www.ebay.com/itm/EPOXY-RESIN-PERMANENT-GLUE-NON-SAGGING-STRUCTURAL-STRENGTH-MARINE-GRADE-1-GALLON-/220594244713

As one of the strongest dry adhesives, the CNT adhesive was also compared with conventional epoxy permanent adhesives (Supplementary Table 3). Unlike the epoxy permanent adhesive that is non-conducting and often surface specific due to its chemical crosslinking nature, our CNT adhesive can be reversibly used with various surfaces for electrical and thermal managements with a record high adhesion strength among dry adhesives. Although the adhesion strength of the CNT adhesives is not as high as that of the epoxy permanent adhesives, the good electrical and thermal conductivities and the thermal-enhancement of adhesion strength discovered in this study provide additional advantages for the CNT adhesives to be used for various potential applications as either permanent or reversible (particularly at relative low adhesion strengths) adhesives.

Supplementary Discussions

As demonstrated by SEM observation (Fig. 2), the nodes guided the penetration into cavities on rough surfaces and then yielded side contact with complex profiles, which presented a greater real contact area for bonding than on a smooth surface. When the roughness of adhering surface increased with increasing temperature (Supplementary Figs. 10&13), the aligned body of CNT bundles was found to be embedded in a deeply-pitted profile with several times the apparent area on the planar surface, like an interlocked “screw”, which requires a substantial increase in the work for the detachment.

To verify the enhanced adhesion through this CNT nano-interlocking mechanism, we estimated the extra work to pull the CNT bundles out of pits. The VA-DWNT bundle was modeled as an elastic strand to be embedded in a surface asperity, which was assumed as a cylindrical rigid hole with depth of l and radius of a for simplicity, being subjected to pull-out force F (inset in Fig. 3c). Under the loading F, detachment occurred along the pit depth (l) while Δx represents the detachment region.

The strain energy gained in deformation ($W_{\Delta x}$) should be the total energy (W_F) supplied by the loading F mins with the energy available for breaking the adhesion (W_A) taken away, which can be expressed as

$$W_{\Delta x} = W_F - W_A \tag{1}$$

For debond propagation, the energy gained from the extension strain ($W_{\Delta x}$) needs to be equal or exceeding the energy available for breaking the adhesion (W_A), that is,

$$W_{\Delta x} \geq W_A \text{. Hence, } W_F \geq 2W_A \tag{2}$$

W_F can be expressed as $W_F = F\Delta x\varepsilon$, while ε is the strain of CNT bundles subjected to the loading F given by $\varepsilon = \frac{F}{\pi a^2 E}$ (E is the Young's modulus of CNT bundle). Thus, $W_F = F\Delta x\varepsilon = F\Delta x \frac{F}{\pi a^2 E} = \frac{F^2 \Delta x}{\pi a^2 E}$ (3)

By using G_a as the characteristic energy required for detachment of the adhering CNT per unit of bonded area, W_A can be expressed as

$$W_A = 2\pi a \Delta x G_a \tag{4}$$

At the critical condition of the entire CNT bundles being pulled out ($\Delta x \rightarrow l$) under the failure force F_f, $W_{F_f} = 2W_A$ (5)

From Supplementary Equations 3 and 4, the Supplementary Equations 5 ($W_{F_f} = 2W_A$) can be translated as

$$\frac{F_f^{\,2} \Delta x}{\pi a^2 E} = 4\pi a \Delta x G_a \qquad (\Delta x \rightarrow l) \tag{6}$$

The energy balance equation can be written as

$$F_f^{\,2} = 4\pi^2 a^3 E G_a \tag{7}$$

Then Supplementary Equations 3 at the critical failure force ($\Delta x \rightarrow l$) can be written as

$$\mathrm{W}_{F_\mathrm{f}} = \frac{F_\mathrm{f}^2 \Delta x}{\pi a^2 E} = \frac{(4\pi^2 a^3 E\mathrm{G_a})\Delta x}{\pi a^2 E} = 4\pi a \Delta x \mathrm{G_a} \approx 4\pi a l \mathrm{G_a} \qquad (8)$$

which shows the additional energy required to pull a CNT bundle out from the deep pit is 2 times that was expected based on the vdW contact with the additional bonded area. The stretching deformation of CNTs during detachment should also be the main contribution to the additional pulling-out energy, arising from the adhesion enhancement through nano-interlocking.

It is supposed that there are n holes per unit area of the surface, then the extra work $\mathrm{W}=\mathrm{nW}_1$ while the area bonded in the normal side contact is $1-\mathrm{n}\pi a^2$. The increased work of detachment per unit of apparent area of roughened surface at the different temperatures can be expressed as:

$$\mathrm{G_a(T)} = (1-\mathrm{n}\pi a^2)\mathrm{G_a} + 4\mathrm{n}\pi a l(\mathrm{T})\mathrm{G_a} \qquad (9)$$

Taking φ (ranged from 0 to 1) for the fractional area of holes with CNT strand embedded in per unit area of surface, where $\varphi = \mathrm{n}\pi a^2$, then the enhancement factor at a specific temperature (T) can be expressed as normalization of the pull-out work associated with the asperities at T defined by l(T) to the corresponding peel-off force at room temperature (24°C) as in Equation 1 in the main text.

Supplementary References

1. Qu, L., Dai, L., Stone, M., Xia, Z. & Wang, Z. L. Carbon nanotube arrays with strong shear binding-on and easy normal lifting-off. *Science* **322,** 238-242 (2008).

2. Qu, L. & Dai, L. Gecko-foot-mimetic aligned single-walled carbon nanotube dry adhesives with unique electrical and thermal properties. *Adv. Mater.* **19,** 3844-3849 (2007).

3. Sethi, S., Ge, L., Ci, L., Ajayan, P. M. & Dhiojwala, A. Gecko-inspired carbon nanotube-based self-cleaning adhesives. *Nano Lett.* **8,** 822-825 (2008).

4. Zhao, Y. et al., Interfacial energy and strength of multiwalled-carbon-nanotube-based dry adhesive *J. Vac. Sci. Technol. B* **24,** 331-335 (2006).

5. Rong, Z. et al., Bio-Inspired herarchical polymer fiber–carbon nanotube adhesives. *Adv. Mater.* **26,** 1456-1461 (2014).

6. Li, Y. et al., Adhesion performance of gecko-inspired flexible carbon nanotubes dry adhesive, *Proc. SPI,* **8686,** 86860S-4 (2013).
